# CAN SUCRALFATE ENEMA PREVENT COLITIS IN COLONIC SEGMENTS WITHOUT FECAL TRANSIT?

**DOI:** 10.1590/0102-672020210002e1630

**Published:** 2022-01-31

**Authors:** Roberta Laís Silva MENDONÇA, Danilo Toshio KANNO, José Aires PEREIRA, Fabio Guilherme CAMPOS, Camila Morais Gonçalves da SILVA, Bruna Zini de Paula FREITAS, Carlos Augusto Real MARTINEZ

**Affiliations:** 1 Laboratório de Investigação Médica, Programa de Pós-Graduação em Ciências da Saúde, Universidade São Francisco, Bragança Paulista, SP, Brasil; 2 Departamento de Gastroenterologia, Faculdade de Medicina, Universidade de São Paulo, São Paulo, SP, Brasil; 3 Departamento de Farmacologia, Centro Universitário da Uni Metrocamp, Campinas, SP, Brasil; 4 Departamento de Cirurgia, Universidade Estadual de Campinas - Unicamp, Campinas, São Paulo - SP, Brasil.

**Keywords:** Colitis, Claudin-3, Occludin, Image processing, Computer-assisted, Sucralfate, Colite, Claudina-3, Ocludina, Processamento de imagem assistida por computador, Sucralfato

## Abstract

Oxidative stress is one of the main mechanisms associated with the rupture of the defense mechanisms of the colonic epithelial barrier; it reduces the tissue content of the claudin-3 and occludin proteins, which are the main constituents of intercellular tight junctions. Sucralfate (SCF) has antioxidant activity and has been used to treat different forms of colitis.

**AIM:**

This study aimed to measure the tissue claudin-3 and occludin content of the colon mucosa without fecal transit, subjected to intervention with SCF.

**METHODS:**

Thirty-six rats were subjected to left colon colostomy and distal mucous fistula. They were divided into two groups according to euthanasia that was performed 2 or 4 weeks after the intervention. Each group was divided into three subgroups according to the enema applied daily: saline alone, SCF at 1 g/kg/day, or SCF at 2 g/kg/day. Colitis was diagnosed by the histological analysis adopting the previous validate scale. The tissue expression of both proteins was identified by immunohistochemical technique. The content of proteins was quantified by computer-assisted image analysis.

**RESULTS:**

The inflammatory score was high in colonic segments without fecal transit, and enemas with SCF reduced the inflammatory score in these segments, mainly in those animals submitted to intervention with SCF in greater concentration and for a longer period of intervention. There was an increase in tissue content of claudin-3 and occludin, related to SCF concentration. The tissue content of both proteins was not related to the intervention time.

**CONCLUSION:**

Enemas with SCF reduced the inflammation and increased the tissue content of claudin-3 and occludin in colonic mucosa without fecal stream.

## RESUMO - RACIONAL

O estresse oxidativo é um dos principais mecanismos associados à ruptura dos mecanismos de defesa que formam a barreira epitelial cólica e reduz o conteúdo tecidual das proteínas claudina-3 e ocludina principais constituintes das junções de oclusão intercelulares. O sucralfato, possui atividade antioxidante e tem sido usado para tratar diferentes formas de colite.

**OBJETIVO:**

Mensurar o conteúdo tecidual de claudina-3 e ocludina da mucosa do cólon sem trânsito fecal, submetido à intervenção com sucralfato.

**MÉTODO:**

Trinta e seis ratos foram submetidos à colostomia do cólon esquerdo e fístula mucosa distal. Os animais foram divididos em dois grupos de acordo com a eutanásia ser realizada duas ou quatro semanas após a intervenção. Cada grupo foi dividido em três subgrupos de acordo com o tipo de intervenção realizada diariamente: solução salina isolada; sucralfato a 1 g/kg/dia ou sucralfato a 2g/kg/dia. A colite foi diagnosticada por análise histológica adotando escala de validação prévia. A expressão tecidual de ambas as proteínas foi identificada por imunoistoquímica. O conteúdo das proteínas foi quantificado por análise de imagem assistida por computador.

**RESULTADOS:**

O escore inflamatório foi maior nos segmentos cólicos sem trânsito fecal e os enemas com sucralfato reduziram o escore inflamatório nesses segmentos, principalmente nos animais submetidos à intervenção com sucralfato em maior concentração e por período mais longo de intervenção. Houve aumento no conteúdo tecidual das proteínas claudina-3 e ocludina, relacionado com a concentração de sucralfato. O conteúdo tecidual de ambas as proteínas não se modificou com a duração da intervenção.

**CONCLUSÃO:**

Enemas com sucralfato reduzem a inflamação e aumentam o conteúdo tecidual de claudina-3 e ocludina na mucosa cólica sem trânsito intestinal.

## INTRODUCTION

The colonic epithelium is the most important defensive barrier of the human body^1^. It consists of only a single layer of specialized cells and forms a highly dynamic and selective barrier that controls the absorption of fluid and solutes by restricting pathogen access to underlying tissues^7^ ^,^ ^31^. The cells of the colonic epithelium must sense and respond appropriately to the constant immunological challenge of the colonic luminal contents and, at the same time, need to allow absorption of water, nutrients, and molecules important for maintaining the cellular energy metabolism^1^. This efficient barrier function is achieved by a series of intercellular junctions that include apical tight junctions (TJs) and subjacent adherent’s junction, desmosomes, and gap junctions, which mediate intercellular adhesion and the communication between adjacent epithelial cells^14^. The mucus layer covers the colonic epithelium, the cytoplasmatic membrane of the cells that forms the colic glands, and basal membrane; immunoglobulins, cytokines, and leukocytes form the immune barrier against pathogens and participate in this mechanism of defense^14^ ^,^ ^22^.

The TJs are the most apical component of the intercellular junctions’ systems and provide an efficient form of cell-cell adhesion in colonic epithelium^34^. They connect adjacent cells together to determine controlled paracellular permeability through the lateral intercellular space^34^. Increasing importance is being attributed to TJs in the mechanisms of cell proliferation, production of mucus, identification of antigens and pathogenic bacteria, and production of antimicrobial peptides to ensure effective immune cell differentiation^27^. TJs are composed of multiple proteins such as claudins family, occludin, tricellulin, and junctional adhesion molecule^15^.

Mucosal inflammation as observed in inflammatory bowel disease compromises the epithelial barrier, resulting in the exposure of lamina propria tissue compartments to luminal antigens and microbes, thus contributing to the inflammatory response and epithelial-barrier defects^7^ ^,^ ^8^ ^,^ ^12^. An experimental study showed that in diversion colitis (DC), an inflammatory disease occurs in colonic segments devoid of the fecal stream, and the TJs are compromised, leading to a rupture of the epithelium barrier^8^ ^,^ ^27^. It is possible that the increased production of reactive oxygen species by epithelial cells deprived of a normal supply of short-chain fatty acids, that is, the main energy substrate to normal metabolism of these cells, is one of the possible mechanisms related to the breakdown of the proteins constituting the TJs^4^ ^,^ ^9^ ^,^ ^27^. This possibility is reinforced by the results of studies showing that the application of enemas with various substances with antioxidant activity, or solutions rich in short-chain fatty acids can improve the inflammatory process of colonic mucosa excluded from the fecal stream and reestablish the different mechanisms of defense that form the epithelial barrier^15^ ^,^ ^21^ ^,^ ^23^ ^,^ ^35^.

Sucralfate (SCF) is a cell-protective agent that has been used for more than three decades in the treatment of duodenal peptic ulcers and reflux esophagitis^29^. The substance is a sucrose and sulfate-aluminum complex, which, in contact with the inflamed mucosa of the gastrointestinal tract, adheres tightly to proteins on the surface of ulcerations, mainly albumin and fibrinogen, thus forming a stable and insoluble complex, creating a protective layer that covers, and protecting the epithelial damage^20^ ^,^ ^29^. Studies have shown that SCF can be used with success in radiation proctitis^20^ ^,^ ^28^. Recently, it was shown that the use of high concentrations of SCF decreases the production of reactive oxygen species and improves the mucosal healing in the experimental models of DC showing that the substance has antioxidant activity^19^. Enemas with SCF, either alone or in association with other drugs, have shown efficacy for the treatment of inflammatory bowel disease and DC^2^ ^,^ ^10^ ^,^ ^18^ ^,^ ^24^. However, to the best of our knowledge, no study has evaluated the effectiveness of the application of enemas containing SCF in the tissue content of the proteins claudin-3 and occludin in colonic epithelium devoid of the fecal stream. It is possible that SCF, due to its antioxidant activity, can protect the TJs from the harmful action of reactive oxygen species.

Thus, the objective of this study was to quantify the tissue content of claudin-3 and occludin proteins in the colonic mucosa devoid of fecal stream submitted to the daily application of SCF enemas in two different concentrations for 2 or 4 weeks.

## METHODS

This study was performed in accordance with the Brazilian Federal Law No. 11,794 and the guidelines of the Brazilian College for Animal Experimentation (COBEA). This experimental study was approved by the Research Ethics Committee, São Francisco University, Bragança Paulista - SP, Brazil (process nº. 2211/07).

### Surgical technique: diversion of the fecal transit

The surgical methodology used for the induction of exclusion colitis has already been described in the previous studies^2^ ^,^ ^5^. In brief, all animals were placed under general anesthesia by the intramuscular administration of 0.1 ml/100 g of a 1:1 (v/v) ketamine (50 mg/ml) and xylazine (20 mg/ml). The abdominal wall was open by a 5-cm midline incision, the left colon 8 cm above of anal margin was sectioned, and the cranial segment was exteriorized as a proximal colostomy. The distal segment of the sectioned left colon was catheterized with a polyvinyl catheter, and it was irrigated with saline until the effluent drained through the animal’s anus no longer contained fecal material. After irrigation, the catheter was removed, and the distal segments of the colon were exteriorized as a distal colostomy. The abdominal incision was closed in two layers. During the postoperative period, the rats were maintained in individual cages without particular care for the stomas or abdominal incisions. Analgesia was improved by diluting dipyrone (15 mg/kg) into the water offered daily and the antibiotic that is not used. After surgery, the animals were kept in individual cages for a period of 6 weeks for the development of DC. This same period was adopted in the previous studies^30^.

### Experimental groups

A total of 32 Wistar rats were divided into three groups with 12 rats in each. The intervention with the proposed solutions was initiated 6 weeks after the surgery of derivation of the fecal stream. The first group received daily enemas containing saline. The second and third groups received daily enemas containing SCF (EMS Sigma Pharma Ltd., Brazil) at two different concentrations (1 and 2 g/kg/day, respectively). In each group, six animals were sacrificed after 2 weeks, and the other six after 4 weeks after the intervention.

### Sample collection

On completion of the intervention period, the rats were anesthetized as described above, and the midline incision was opened again. In both groups, specimens were taken from the colon without fecal stream subjected to irrigation with saline and SCF at both concentrations. It removed a specimen of the colon without fecal stream with 4.0 cm of length. To standardize the histological analyses, in all animals, these segments of the colon without fecal stream were always removed 0.5 cm above the Peyer’s lymphoid plaque. Then, the specimens were opened through the anti-mesenteric border fixed in a piece of cork and referred to histological and immunohistochemical techniques. The euthanasia was performed by intracardiac injection of the lethal dose of thiopental.

### Histological analysis

The colon specimens without fecal stream removed for histological analysis were immersed in 10% neutral formalin buffer for 24 h and then dehydrated by exposure to increasing ethanol concentrations, xylene, and embedded in paraffin. Thereafter, the sections of tissue were cut at 5 μm and were mounted on a glass slide, cleared, hydrated, and stained with H&E for the evaluation of the presence of colitis and the degree of inflammation. The slides were analyzed under an optical microscope (Eclipse F-50, Nikon Inc., Osaka, Japan) at a magnification of 200×. The slides prepared for a pathologist who was unaware of the objectives of the study evaluated both histology and immunohistochemistry (anti-claudin-3 and anti-occludin).

Photomicrographs were taken with a digital video-capture camera (DS-Fi-50; Nikon Inc., Osaka, Japan) coupled to the microscope body. The presence of colitis in the colon segments devoid of the fecal stream was confirmed considering three different histological parameters: mucosal-submucosal neutrophil infiltration, presence of epithelial erosion and ulceration, and classified in crosses (- to 9+) for each variable. The severity of the inflammation in the colonic mucosa devoid from the fecal stream was established in accordance with a previously used inflammatory grade scale^4^.

### Immunohistochemical staining

For the immunoexpression study of claudin-3 and occludin proteins, we used standardized methodology adopted in other studies and obeyed the datasheet of the manufacturers of each of the primary antibodies^17^. As primary antibody, we used anti-claudin-3 monoclonal antibody (Ref. E-3834, Lot. 110520, Spring Bioscience, Pleasanton, CA, USA). The anti-claudin-3 primary antibody was mixed 1:50 in bovine serum albumin (1%). The monoclonal antibody anti-occludin (Ref. E-17464, Lot. 111207S, Spring Bioscience) was mixed 1:100 in bovine serum albumin (1%). The slides were covered with approximately 100 μL of these solutions and were incubated at 4°C for 24 h. After exposure to the primary antibody, the slides were washed with phosphate-buffered saline (PBS) and incubated with a secondary antibody (Lot: H1011 Histofine Code: 414191N, Spring Bioscience). Later, they were incubated with the streptavidin-biotin-peroxidase complex (ABC Staining System, Dako A/S, Glostrup, Denmark). The chromogenic reaction was developed with a freshly prepared solution of diaminobenzidine tetrahydrochloride (DAB, 10 mg in 10 ml PBS). The slides were washed and counterstained with methyl green for 1 min and washed again in distilled water. Then, the slides were dehydrated by immersion in the crescent concentration of ethanol followed by xylene. Finally, they were mounted, labeled, and kept in a horizontal position for 24 h.

Immunostaining was considered positive when a diffuse brownish color with spots of varying intensity and homogeneous distribution in the apical or basolateral cellular membrane was observed. As recommended by the datasheet of both primary antibodies used, a negative control was prepared without the addition of the primary antibody, and a positive control for claudin-3 and occludin was prepared using normal human small bowel tissue, which is known to be positive for both proteins.

### Image processing, computer assisted

The tissue content of claudin-3 and occludin was quantified by means of computerized morphometry and was always performed in a focal field in which there were at least three complete and contiguous colonic glands. These images were analyzed using the NIS-Elements version 3.1 software (Nikon Inc., Osaka, Japan). The software would transform the color intensity in the number of pixels in each field selected. The pixel values were transformed into the percentage of protein expressions by analyzed fields (%/fields). The final value taken for each field measured in the colonic segments was the mean of the values found from evaluating three different fields.

### Statistical analysis

The statistical analysis was performed by taking the significance level of 5% (p<0.05). The data from each colon segment analyzed, in each experimental group, were expressed as the mean value with the respective standard error and were analyzed using the Biostat version 5.0 for statistical software. To compare the grade of inflammatory score among the different experimental subgroups, the nonparametric Mann-Whitney U test was used. To compare the content of claudin-3 and occludin among the different experimental subgroups, the Student’s *t*-test was used. To analyze the variance in the claudin-3 and occludin tissue content among the different experimental groups, analysis of variance (ANOVA) was used.

## RESULTS

**Figure 1 -:**
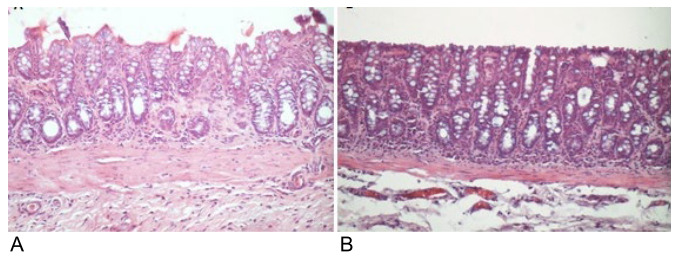
(A) Colonic epithelium devoid of fecal stream submitted to intervention with saline for 4 weeks. (B) Colonic epithelium devoid from fecal stream submitted to intervention with SCF at a concentration of 2.0 g/kg/day.

Figure 1 shows colonic segments devoid of the fecal stream in animals submitted to intervention with saline and SCF at a concentration of 2.0 g/kg/day. Animals submitted to intervention with saline present more epithelial damage when compared to those treated with enemas containing SCF at a concentration of 2.0 g/kg/day.

**Figure 2 -:**
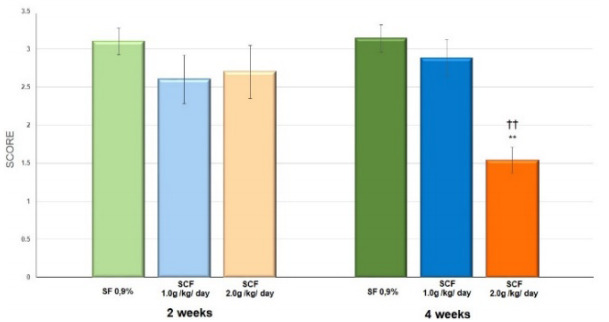
Mean values of the inflammatory grade score found in animals submitted to intervention with saline solution, SCF 1.0 and 2.0 g/kg/day, for 2 and 4 weeks. **Significant: SCF 2.0 g/kg/day × saline (p<0.01); ††Significant: SCF 2.0 g/kg/day × SCF 1.0 g/kg/day (p<0.01). Mann-Whitney U test.

Figure 2 shows the inflammatory grade score, comparing colonic segments without fecal stream submitted to intervention with saline, SCF 1.0 and 2.0 g/kg/day, by 2 or 4 weeks. The inflammatory grade score decreases in animals submitted to intervention only when was employed high concentration of the drug and for a longer period of intervention.

**Figure 3 -:**
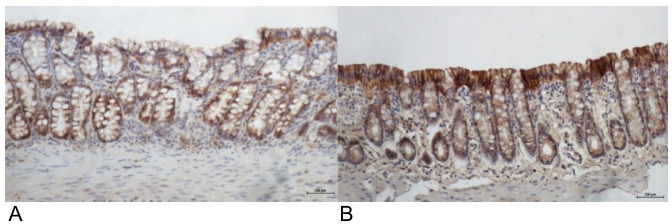
(A) Colonic epithelium without the fecal stream of animals submitted to intervention with saline for 4 weeks, with a loss of expression of claudin-3 on the epithelial surface with the formation of ulcers (IH 200×). (B) colonic epithelium without fecal stream after intervention with SCF 2.0 g/kg/day for 4 weeks with an increase of expression of the claudin-3 protein in the apical portion of the colic glands (IH 200×).

Figure 3 shows the tissue expression of claudin-3, comparing colonic segments without fecal stream submitted to intervention with saline and SCF at a concentration of 2.0 g/kg/day, by 4 weeks.

**Figure 4 -:**
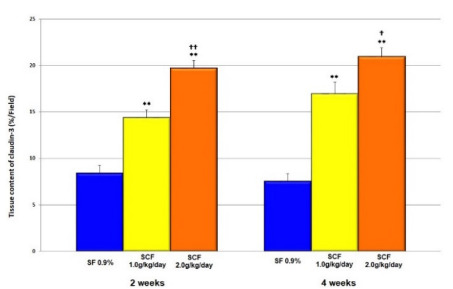
Mean values of tissue content of claudin-3 found in animals submitted to intervention with saline, SCF 1.0 and 2.0 g/kg/day, for 2 and 4 weeks. **Significant: SCF 1.0 g/kg/day × saline and SCF 2.0 g/kg/day × saline (p<0.0001); †Significant: SCF 2.0 g/kg/day × SCF 1.0 g/kg/day (p=0.01); ††Significant: SCF 2.0 g/kg/day × SCF 1.0 g/kg/day (p=0.0003). Student’s t test.

Figure 4 compares the tissue content of claudin-3 in colonic segments without fecal stream submitted to intervention with saline, SCF 1.0 and 2.0 g/kg/day, by 2 or 4 weeks. The tissue content of claudin-3 increases in animals submitted to intervention with SCF, independent of the concentration or time of intervention. However, in animals submitted to intervention with a high concentration of SCF, the tissue content of claudin-3 increases so much.

**Figure 5 -:**
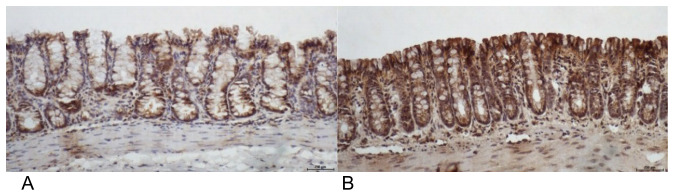
(A) Colonic epithelium without the fecal stream of an animal submitted to intervention with saline for 2 weeks, with loss of occludin expression on the epithelial surface with the formation of ulcers (IH 200×). (B) colonic epithelium without the fecal stream after intervention with SCF 2.0 g/kg/day for 4 weeks with an increase of the expression of the protein occludin in the apical and basolateral portion of the colonic glands (IH 200×).

Figure 5 shows the tissue expression of occludin, comparing colonic segments without fecal stream submitted to intervention with saline and SCF at a concentration of 2.0 g/kg/day, by 2 or 4 weeks.

**Figure 6 -:**
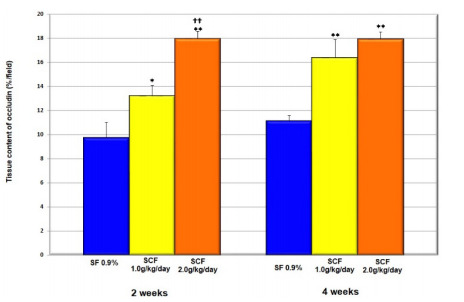
Mean values of occludin tissue content found in animals submitted to intervention with saline, SCF 1.0 and 2.0 g/kg/day, for 2 and 4 weeks. **Significant: SCF 1.0 g/kg/day × saline and SCF 2.0 g/kg/day × saline (p<0.0001); ††Significant: SCF 2.0 g/kg/day × SCF 1.0 g/kg/day (p=0.0003). Student’s *t*-test.

Figure 6 compares the tissue content of occludin in colonic segments without fecal stream submitted to intervention with saline, SCF 1.0 and 2.0 g/kg/day, by 2 or 4 weeks. The tissue content of occludin increases in animals submitted to intervention with SCF, independent of the concentration or time of intervention.

There was no variation in the tissue content of claudin-3 and occludin related to the intervention time (2 or 4 weeks), in the animals submitted to intervention with saline, SCF 1.0 or 2.0 g/kg/day.

## DISCUSSION

The SCF is the salt formed by the sucrose octasulfate disaccharide associated with polyaluminium hydroxide^13^ ^,^ ^33^. The substance is considered a cytoprotective complex and is initially used to prevent or treat diseases of the upper digestive tract, mainly represented by peptic ulcer disease, stress ulcers, and acute erosions of the gastric mucosa^29^ ^,^ ^33^. Subsequently, due to its ability to adhere to erosions of the inflamed epithelium, SCF also proved effective in the treatment of patients with radiation-induced proctitis and those with inflammatory bowel disease, particularly distal erosive proctitis^3^ ^,^ ^10^ ^,^ ^13^ ^,^ ^28^. Since then, a series of authors have published the results of using SCF for the treatment of different colic diseases that evolve with inflammation, such as ulcerative colitis and radiation proctitis^25^ ^,^ ^26^. However, reviewing the literature, no study has evaluated the efficiency of SCF in patients with DC, and only our group has been studying the effectiveness of SCF in an experimental model of it^2^ ^,^ ^6^ ^,^ ^18^ ^,^ ^24^. These studies showed that daily enemas with SCF reduce the inflammatory infiltrate and the oxidative damage in colonic mucosa devoid of the fecal stream and increase the tissue content of several types of mucins that recover the epithelium and improve the healing of the colonic epithelium^2^ ^,^ ^18^ ^,^ ^19^ ^,^ ^22^ ^,^ ^24^. Probably, all of these results are related to the SCF’s ability to stimulate the production of mucus by the epithelial cells of the gastrointestinal mucosa, to increase the synthesis of the epithelial growth factor improving the epithelial healing, and, particularly, by its antioxidant and anti-inflammatory action^19^ ^,^ ^29^.

The colonic epithelium acts as a morphological and functional barrier because, despite having selective permeability, it guarantees protection against the invasion of harmful agents present in the intestinal lumen^31^ ^,^ ^32^ ^,^ ^34^. This is achieved through multiple defense mechanisms involving various cell types-epithelial and nonepithelial-that work in an integrated manner to build protective barriers at mucosal sites^24^ ^,^ ^29^ ^,^ ^30^. One of the most important of these mechanisms of defense is represented by intercellular junctions’ systems, particularly by TJs. Studies in experimental models of induced colitis and in inflammatory bowel disease patients have shown that the breakdown of intercellular junctions is an early event in the etiopathogenesis of the disease^8^ ^,^ ^11^ ^,^ ^14^. Oxidative stress has been shown to be one of the main mechanisms involved in breaking down these intercellular junction systems^9^ ^,^ ^12^. Studies have shown that these junctions are compromised in different forms of colitis and that short-chain fatty acids deficiency can cause a break of the intercellular junctions^32^. The integrity of intercellular junctions was studied in an experimental model of DC^16^ ^,^ ^17^. In these studies, it was measured the tissue content of the main proteins that comprise the TJs (claudin-3 and occludin) compared to the colon segments provided and devoid from the fecal stream. It was found that there was a marked reduction in the content of both proteins in the cells of the gland of the colic mucosa devoid of intestinal transit^16^. This reduction was more accentuated in the content of claudin-3, the main protein that constitutes the TJs of the colonic mucosa^16^. The reduction in the tissue content of both TJ proteins was inversely related to the levels of oxidative stress and the worsening of tissue inflammation^16^. The application of enemas containing substances with high antioxidant activity, such as oily extract of curcumin, increases the tissue content of claudin-3 and occludes colonic mucosa devoid of the fecal stream^17^.

When considering that reactive oxygen species can lead to breaking of TJs in experimental models of DC and that SCF, in addition to antioxidant properties, can protect the intestinal epithelium by increasing mucus production and favoring epithelial healing, it would be interesting to assess the substance’s efficiency in preserving the TJs in a DC model^2^ ^,^ ^4^ ^,^ ^5^ ^,^ ^6^ ^,^ ^18^ ^,^ ^24^. The results of this study show that the tissue content of both proteins increases in the colonic segments devoid of the fecal stream submitted to the intervention with SCF, independent of the concentration used and the time of intervention. However, when SCF was used at a concentration of 2.0 g/kg/day, the maintenance of tissue content of both proteins is most significant. At this concentration, the inflammatory grade score also reduces significantly, confirming the anti-inflammatory properties of the substance. Previous studies also show that intervention with SCF increases the tissue content of neutral mucins, total acid mucins, sulfomucins, sialomucins, and MUC-2, related to the reduction in the inflammatory intensity^2^ ^,^ ^4^ ^,^ ^5^ ^,^ ^6^ ^,^ ^18^ ^,^ ^24^. It is likely that the antioxidant and anti-inflammatory action of SCF, demonstrated in previous studies, may be the main protection mechanism for TJs^2^ ^,^ ^5^.

The results of this study suggest that SCF may be a useful therapeutic strategy for the treatment of DC. As the drug has a low cost and good availability, it is possible that it can be used in patients with DC where the possibility of reestablishing fecal transit is not envisaged. However, studies in humans, with a larger number of patients and with longer follow-up, are still needed to confirm these perspectives.

## CONCLUSION

Enemas with SCF reduce the inflammation and increase the tissue content of claudin-3 and occludin in colonic segments devoid of the fecal stream in an experimental model of DC.
